# Work Performance, Mood and Sleep Alterations in Home Office Workers during the COVID-19 Pandemic

**DOI:** 10.3390/ijerph19041990

**Published:** 2022-02-10

**Authors:** Chiara Costa, Michele Teodoro, Carmela Mento, Federica Giambò, Carmen Vitello, Sebastiano Italia, Concettina Fenga

**Affiliations:** 1 Clinical and Experimental Medicine Department, University of Messina, 98125 Messina, Italy; ccosta@unime.it; 2 Department of Biomedical and Dental Sciences and Morphofunctional Imaging, Occupational Medicine Section, University of Messina, 98125 Messina, Italy; michele.teodoro@unime.it (M.T.); federicagiambo@gmail.com (F.G.); carmenvitello821@gmail.com (C.V.); cfenga@unime.it (C.F.); 3 Department of Biomedical and Dental Sciences and Morphofunctional Imaging, Clinical Psychology, Psychiatric Unit, University of Messina, 98125 Messina, Italy; carmela.mento@unime.it

**Keywords:** COVID-19, home office workers, work perception, mood, sleep quality

## Abstract

The sudden burst of the COVID-19 pandemic has changed the work environment in favor of remote working, affecting the perception of work quality, satisfaction and performance. This crisis has also influenced workers’ mood, sleep quality and general perception of everyday life. Our main purpose in this study was to give empirical contributions about home office workers experiencing remote working during the pandemic by assessing mood spectrum variations, sleep disturbances and the general impact of pandemic in everyday life. This cross-sectional study was performed between November and December 2020 through an online questionnaire. Participants were office workers performing remote work from workstations settled at home. The questionnaire investigated sociodemographic characteristics, health factors, perception of remote working, mood spectrum, sleep quality and pandemic context perception. The sample consisted of 94 respondents: 63 women and 31 men; the mean age was 50.4 years. Study population showed great satisfaction for remote working performance and online services for video connections. Only one third of the participants reported higher levels of irritability and loneliness and 16% of women complained of nightmares. Most of participants stated that the pandemic importantly affected daily life (85.1%). Half of female subjects with children <18 years stated that children’s age influenced their work performance. Since the pandemic is still an ongoing issue, the lesson learnt is that local government actions are needed to assist home office workers through tailored programs to support families. Given the central role of women in childcare, female workers would mainly benefit from social support accordingly to their parental tasks and remote work organization.

## 1. Introduction

The sudden burst of the COVID-19 pandemic and the subsequent preventive measures, such as quarantine, social isolation, partial or total lockdown, not only have dramatically transformed everyday life, with a massive impact on physical, mental, social, and financial welfare of the population [1] but they have also changed the work environment, in favor of remote work organization. Subsequently, the perception of work quality, satisfaction and performance were all affected [2]. The output obtained by entering the search keywords “remote work” in the main databases showed an exponential increase in the number of scientific surveys in the years 2019–2021.

The revolutionary changes faced by people, communities and governments during the COVID-19 pandemic outbreak have totally restructured work organizations [1,2]. Enforced remote working, staff reduction, temporary assembly stoppage are some events that disturbed work processes, distribution of responsibilities, job pressures and performances [3]. These unforeseen changes have forced workers, whose children were in preschool or remote school-age, to adapt their working schedule in order to accomplish all caregiving responsibilities in contrast to those workers living alone or without children. Workers with parental responsibility were suddenly and personally involved in childcare during the whole day, therefore, the new home workspaces have been characterized by noise, discomfort and loss of privacy, possibly affecting workers’ performance [4]. Workers and employers were completely unprepared to handle this psychological, emotional and social burden which required achieving new organizational skills [5]. It is worthwhile to consider the role of managerial functions, aiming not only to adopt interventions planned in personnel management (enrolment, training, benefit assignment, information sharing) but also on workers’ perceptions and performances that are strongly linked to organizational flexibility [6]. Since human capital is the most valuable investment of the organizations, this crisis led to very important consequences in the workforce management, by negatively influencing workers’ perceptions and approaches towards the work environment [7].

Previous researches explored employees’ work perception regarding levels of satisfaction, enthusiasm, work commitment, perceived structural support and organizational performances [8,9,10,11]. During the COVID-19 pandemic, work organizations have been required to use unexpected measures, such as implementation of remote working, reduction of working hours and even dismissals, profoundly impacting workers’ perceived job insecurity and social isolation [12,13].

Although most studies agreed in assuming that workers have a great responsibility in determining failure or success within work cycle processes, employees are generally quite hesitant towards organizational changes [14,15]. In fact, these innovations might be related to a rise in workloads, new assigned tasks, adjustment in work relationships and new tactical objectives [16]. Nevertheless, when the organization positively introduces work changes, employees could perceive this process as an opportunity for personal and occupational growth, thus establishing constructive attitudes and optimistic effects on job performance [17,18]. Consequently, workers could be involved in the organizational transition plans in order to create favorable emotions and mindsets toward these collective efforts [19].

Under these premises, our main purpose was to give empirical contributions through the discussion of results coming from an online survey conducted among home office workers experiencing remote working during the pandemic. In particular, the investigation evaluated employees’ feelings about remote working, focusing in both quality and performance, along with sleep disturbances, mood alterations and the general impact of the pandemic in everyday life.

## 2. Materials and Methods

### 2.1. Study Design and Population

This cross-sectional study was performed between November and December 2020, through an online questionnaire distributed via social networks. The self-administered questionnaire was composed of thirty-three items and took no more than ten minutes to be completed. The first section investigated sociodemographic characteristics and health factors of the sample: age, gender, educational level, marital status, number of children, chronic physical diseases, chronic medication administration, contacts with SARS-CoV-2 infected subjects and infection risk exposure. The second section consisted in an interview about work perception, mood alterations, occurrence of sleep disturbances, current and future perspective of the pandemic.

Participants were office workers employed by an outsource labor provider in different administrative facilities in Italy; the average annual income level was comprised between EUR 15,000 and EUR 29,000. All the interviewed subjects worked remotely and they had settled their workstation at home. The sampling method included the spreading through internet media of an invitation, including the link to the online survey. The invitation was emailed to office managers with the request to extend it to their teams. A dedicated online platform was used to collect the data from recruited subjects.

This study was carried out in accordance with the Declaration of Helsinki’s ethical standards. Participation was voluntary and without compensation. All the subjects provided informed consent.

### 2.2. Procedures and Measures

The second section of the self-administered questionnaire investigated different aspects of the perception of remote working, mood, sleep disturbances and the pandemic impact on everyday life.

#### 2.2.1. Work Perception and Video Connections

Participants were asked whether they thought that the age of their children affected their work performance (“Yes” or “No”). Respondents were also asked if they were satisfied with their work performance despite the pandemic (“Yes” or “No”), how the work had changed (“the work got worse” or “the work is unchanged” or “the work has improved”) and if they were differently connected to the internet (“less than before” or “as before” or “more than before”). The level of satisfaction regarding the online services for video connections and the sensation of feeling more tired after a video call were measured using a 5-point Likert-type scale, from 1 (never) to 5 (always).

#### 2.2.2. Mood and Sleep Quality

Multiple aspects were investigated and measured with a 5-point Likert-type scale, from 1 (never) to 5 (always), such as: irritability (“do you feel irritable?”), loneliness (“do you feel lonely?”), sleep problems (“are you having problems in falling asleep?” and “do you wake up in the night and have difficulties in getting back to sleep?”). Participants were also asked whether they had nightmares. 

#### 2.2.3. Pandemic Impact on Everyday Life

Subjects reported their perception of the pandemic on everyday life (“do you think that the pandemic has affected your life?”). They also rated their fear of contagion and the will to go out and return to previous life by using a 5-point Likert-type scale, from 1 (not at all) to 5 (very much).

### 2.3. Statistical Analysis

Descriptive analyses were performed for all variables. Categorical variables were expressed as frequency and proportion; continuous variables were expressed as mean and standard deviation. To evaluate differences between women and men in categorical variables, we used chi-square tests and Fisher’s exact tests, as appropriate. After verifying the non-Gaussian distribution in all the continuous variables and outcomes through the application of the Kolmogorov-Smirnov test, differences between genders were evaluated using the Mann–Whitney U test. Furthermore, possible predictors of the considered outcomes in the current investigation were assessed by using different models. In particular, the generalized linear models were used to analyze the answers regarding the changes in work perception, irritability, loneliness, problems in falling asleep, nocturnal awakenings with difficulties in getting back to sleep, fear of contagion and willingness to return to previous life. Whereas binary logistic regression was applied for the assessment of nightmares and pandemic influence on everyday life. The occurrence of associations between the outcomes were assessed through a bivariate correlation with Pearson correlation coefficient. *p* values < 0.05 were considered statistically significant and reported in bold characters in the tables. Beta values of the statistical models were reported in tables as “B” and *p* values as “P”. Statistical analysis was performed using IBM SPSS Statistics 23 (IBM Corp., Armonk, NY, USA).

## 3. Results

A total of 94 respondents (N), 63 women and 31 men, accepted to join the study and completed the assessment. A detailed sample description is summarized in Table 1. 

The mean age of study population was 50.4 years; though women were slightly older than men (51 vs. 48 years, respectively), this difference was not statistically significant. The majority of the individuals possessed a high school diploma; moreover, one third of the female subjects was graduated. Married/cohabitant subjects were similarly represented among women and men (about 65%), but more men than women had no children (32% and 24%, respectively). Only nearly 20% of participants had children under 18 years of age.

About one third of female respondents had physical illnesses (15 hypertension, 3 diabetes, 2 autoimmune disorders and 1 respiratory disease); only 20% of men suffered from a disease (5 hypertension, 1 diabetes) and none of the subjects suffered from mental illnesses. Within the totality of samples, 5 participants had a contact with a SARS-CoV-2 infected subject and 13 respondents declared they had SARS-CoV-2 infection risk exposure.

A description of the scores resulting from the interview is reported in Table 2. Different outcomes were measured with a 5-point Likert-type scale; in these cases, scores were also dichotomized using 2 as cut-off.

Considering the answers concerning how the age of children was affecting working performance, we found a doubled percentage of men (28.6%) compared to women (14.6%), but after stratifying the subjects depending on children’s age (choosing 18 years as cut-off), half of women vs. one third of men having children <18 years considered their age to be an influencing factor on work performance, as well as one third of men and only 1 over 34 women having children ≥18 years, with a statistically significant difference between men and women.

Almost all of the subjects were satisfied with their work performance despite the pandemic period and above three quarters of them were convinced to be more connected than before through the use of internet. In addition, a good degree of satisfaction with video services was also detected (mean ± SD = 4.1 ± 0.8) with only a few subjects feeling more tired after a video call. The majority of individuals stated that their work was not changed (54.3%) and about one third declared a work improvement. Low scores of irritability (mean ± SD = 2.0 ± 1.2) and loneliness (mean ± SD = 1.7 ± 1.1) were registered. Higher scores were found in assessing sleep quality, in particular, problems in falling asleep (mean ± SD = 2.3 ± 1.2), nocturnal awakenings with difficulties in getting back to sleep (mean ± SD = 2.5 ± 1.3) and 10 subjects (all women) complained of having nightmares. Additionally, women reported higher scores than men regarding their fear of being infected with SARS-CoV-2, with a statistically significant difference. The majority of the subjects had a strong willingness to return to previous life and 85% of the participants declared that the pandemic affected their everyday life.

We applied generalized linear model for the answers regarding the changes in work perception, irritability and loneliness (Table 3), problems in falling asleep and nocturnal awakenings with difficulties in getting back to sleep (Table 4), fear of contagion and willingness to return to previous life (Table 5); binary logistic regression was applied for nightmares’ occurrence (Table 4) and pandemic influence on everyday life (Table 5).

Considering the independent variables used in the statistical models, married/cohabitant subjects felt less lonely than single ones. Being graduated acted as a predictive factor of perceiving a work improvement during the pandemic. Subjects who thought that the age of their children had affected remote working performances were related to lower irritability. Participants without physical disease had a higher risk of complaining about nightmares (OR 4.50). The absence of SARS-CoV-2 infection risk exposure acted as a protective factor against irritability. Moreover, being satisfied with personal work performance acted as a predictor of reporting lower levels of irritability and loneliness. More time than before spent on the internet demonstrated to be a predictor of worse work perception, higher irritability, more problems in falling asleep and nocturnal awakenings and higher fear of infection. People that declared to be connected to the internet like before the pandemic showed to have an increased risk factor (OR 4.33; 95% CI from 1.32 to 14.24) for thinking that the pandemic would be affecting their everyday life.

Furthermore, we considered the possibility to find a correlation between the outcomes of the current investigation (Table 6).

An improvement in work perception was related to a lower irritability (r = −0.29) and lower problems in falling asleep (r = −0.27). Irritability was positively associated with loneliness (r = 0.29), mood alterations were related both to problems in falling asleep and to nocturnal awakenings; additionally, loneliness was associated to nightmares (r = 0.24). All the three aspects of sleep quality (problems in falling asleep, nocturnal awakenings and nightmares) were positively associated with each other. The fear of infection was positively related to problems in falling asleep (r = 0.36) and nocturnal awakenings (r = 0.23). The willingness to go out and return to previous life was negatively associated with nightmares (r = −0.15). Subjects who thought that the pandemic had affected everyday life were related to higher irritability (r = 0.24), more problems in falling asleep (r = 0.24) and nocturnal awakenings (r = 0.27).

## 4. Discussion

In the current study, we investigated workers’ feelings about working from home, related to both quality and performance, along with sleep quality, mood alterations and the general impact of the pandemic on everyday life.

Overall, this population study demonstrated great satisfaction for remote working performance. Only one third of the participants reported higher levels of irritability and loneliness and 16% of women complained of having nightmares. Most of the participants stated that the pandemic importantly affected their daily life (85.1%).

Albeit the majority of the population studied declared no changes in work perception, about one third of the subjects reported a work improvement with a high rate (95.7%) of satisfaction in work performance and online services for video connections. In accordance with the existing literature, home-based workers with good access to communication technologies are more efficient and reliable in performing their work, experiencing determination and connectedness, while scarce online connection services might generate frustration, affecting job performance [20]. Additionally, the effects of home life on work should not be underestimated. On one hand, the interaction between working at home and family life may produce more prolific outcomes in home office workers; on the other hand, some authors sustain the hypothesis that domestic responsibilities may negatively affect telework, especially in those who are not able to establish limitations between the two [21]. Childcare may represent a constraint for home-based working [22]. We investigated whether children’s age influenced work performance. After stratifying the sample in accordance to children’s age (18 years as cut-off), men claimed that children’s age has affected work from home independently from age (almost one third); conversely, half of female subjects with children <18 years complained of a negative impact on work performance from home compared to only one woman with children >18 years. Women are more directly involved in childcare, therefore they may consider young children’s age to be a detrimental factor affecting work performance. These findings might suggest a persistent gender gap both in workplaces and home, resulting in a double burden of work bearing on women.

In our statistical models, being graduated acted as a predictor of an enhancement in work performance. Other authors suggest that the education level may be positively associated with a better perception of work ability [23,24]; since home office workers have been no longer under straight control by their supervisor, we can hypothesize that graduated subjects felt more control and responsibilities, resulting in a better perception in work quality and performance [25]. The interviewed subjects which declared to spend increased time online compared with before the pandemic, showed to have an increased risk of perceiving a worsening in work performance, coupled with higher level of irritability, decreased sleep quality and higher fear of being infected. The subjects were also more driven to think that the pandemic affected their everyday life. Thus, more time spent on the internet has led to worse outcomes, suggesting that an uncontrolled flow of information, the so-called “COVID-19 Infodemic”, may have contributed to negatively affect the perception of remote working, altering mood and sleep quality [26]. Furthermore, our results highlighted that a worsening in work perception has been related to scarce sleep quality and high irritability, probably due to the detrimental effects that a dysfunctional sleep/awake cycle might have on work performance and mood states [27,28,29,30].

In our population study, these mood alterations were predicted by a high SARS-CoV-2 risk exposure. In addition, being not satisfied with remote working performance predicted a higher irritability and loneliness. The perception of the pandemic as a traumatic event may have contributed to modify the mental well-being, resulting in psychological symptoms, and probably leading to increased levels of anxiety and depression [31]. Our results highlighted not only that irritability and loneliness were associated with each other, but also that both were related to sleep insufficiency. The literature suggests that a poor sleep quality can cause tiredness, thereby leading to reduced levels of alertness and amplified irritability [32,33]. Although our statistical models did not find gender as a predictor of poor sleep quality, more women than men (10 vs. 0) complained about nightmares, resulting in a statistically significant gender difference and confirming that women are more susceptible to sleep disturbances [34,35]. 

In our investigation, nightmares are associated with a scarce willingness to go out and to return to the lifestyle conducted before the pandemic. Problems in falling asleep and nocturnal awakenings are related to the idea that the pandemic might have negatively affected the everyday life; this outcome is also related to a higher irritability. Our findings suggested that there could be a mutual interrelation among sleep quality, mental well-being and the perception of the pandemic as a traumatic event, as proposed by other authors [36,37,38].

During the pandemic, remote working increment has brought advantages for the population such as shorter commuting times, greater personal motivation, better work-life balance and better control over schedules. Conversely, it is also important to highlight the disadvantages of home-based working which can be related to employees’ performance checking, communication gaps that may arise or the problems that might be linked with the commixture of domestic with work tasks. Social isolation, satisfaction with video connection services, work-family balance and children’s care are the issues raised in this study. They represent factors which may affect job performance, mood states and psychological well-being, sleep quality and everyday life. It is worthwhile to wonder whether working remotely will become an available option for the majority of office workers, thereby resulting in changes in accommodation and risk assessment, or if it will endure to be seen as a special advantage. This research suggests the need to increase childcare services and to support women with younger children during the acute phases of the pandemic. Finally, particular attention should be paid to the emotional patterns of loneliness and sadness associated with sleep disturbances, which can be configured as subthreshold depressive patterns which need to be managed to further prevent the development of mood disorders.

This study presents some limitations. First, a cross-sectional design does not allow us to establish the direction of causation and to transpose the results to a general population. Secondly, we did not use standardized questionnaires. Moreover, the majority of our sample was composed of female subjects (67%), who tend to be more worried about their health status and are willing to take part in health surveys more often than males [39].

## 5. Conclusions

Our results highlighted that home office workers were satisfied by the perceived work quality and performance; notwithstanding, about a third of the subjects reported mood alterations and women revealed to be more susceptible to sleep disturbances.

The COVID-19 pandemic might be seen as a challenging exam for employers to test their skills in harmonizing a work environment, considering how remote working will progress as the economy evolves in the next few years. 

Since the pandemic is still an ongoing issue, the lesson learnt is that local government actions are needed to assist home office workers through tailored programs to support families. Given the central role of women in childcare, female workers would mainly benefit from social support accordingly to their parental tasks and remote work organization.

It would be also worthwhile to address further research towards the evaluation of the possible amplification of gender inequalities due to working from home.

## Figures and Tables

**Table 1:** Description of study population: sociodemographic characteristics and health status factors.

	Total	Women	Men	*p*-Value
	*n* (%)	*n* (%)	*n* (%)	
Sociodemographic Factors				
Total	94 (100)	63 (67.0)	31 (33.0)	
Age				
Mean ± SD	50.4 ± 7.5	51.6 ± 6.4	48.0 ± 8.9	0.052
Range	30–62	33–62	30–62	
Education				
Middle school	11 (1.7)	7 (11.1)	4 (12.9)	0.600
High School	49 (52.1)	31 (49.2)	18 (58.1)	
Graduation	34 (36.2)	25 (39.7)	9 (29.0)	
Marital status				
Single	33 (35.1)	22 (34.9)	11 (35.5)	0.957
Married/Cohabitant	61 (64.9)	41 (65.1)	20 (64.5)	
Children				
No	25 (26.6)	15 (23.8)	10 (32.3)	0.383
Yes	69 (73.4)	48 (76.2)	21 (67.7)	
<18 years	20 (21.3)	14 (22.2)	6 (19.4)	0.960
≥18 years	49 (52.1)	34 (54.0)	15 (48.4)	
Health Status Factors				
Physical diseases	27 (28.7)	21 (33.3)	6 (19.4)	0.159
Mental illnesses	0 (0.0)	0 (0.0)	0 (0.0)	0.159
Medications	8 (8.5)	6 (9.5)	2 (6.5)	0.616
SARS-CoV-2 infected contact	5 (5.3)	4 (6.3)	1 (3.2)	0.526
SARS-CoV-2 risk exposure	13 (13.8)	8 (12.7)	5 (16.1)	0.651

**Table 2:** Description of the results of the survey regarding work performance, mood and sleep alteration in home office workers during the COVID-19 pandemic, in a gender perspective.

	Total	Women	Men	*p*-Value
	*n*/*N* (%)	*n*/*N* (%)	*n*/*N* (%)	
Do you think the age of your children has affected your working performance?				
Yes ^1^	13/69 (18.8)	7/48 (14.6)	6/21 (28.6)	0.172
Yes (<18 years children) ^2^	8/20 (40.0)	6/14 (42.9)	2/6 (33.3)	1.000
Yes (≥18 years children) ^3^	5/49 (10.2)	1/34 (2.9)	4/15 (26.7)	**0.026**
Are you satisfied with your work performance despite the pandemic?				
Yes	90 (95.7)	60 (95.2)	30 (96.8)	1.000
Do you think you have been connected to the internet (mobile, tablet and PC)…				
Less than before	0 (0.0)	0 (0.0)	0 (0.0)	0.700
As before	22 (23.4)	14 (22.2)	8 (25.8)	
More than before	72 (76.6)	49 (77.8)	23 (74.2)	
Are you satisfied with the online services for video connections?				
≤2	2 (2.1)	2 (3.2)	0 (0.0)	1.000
>2	92 (97.9)	61 (96.8)	31 (100.0)	
Mean (SD)	4.1 (0.8)	4.1 (0.8)	4.0 (0.8)	0.436
Do you feel more tired after a video call?				
≤2	61 (65.6)	43 (68.3)	18 (58.1)	0.364
>2	33 (35.4)	20 (31.7)	13 (41.9)	
Mean (SD)	2.1 (1.3)	2.1 (1.3)	2.2 (1.4)	0.701
Has your work changed?				
The work got worse	16 (17.0)	11 (17.4)	5 (16.2)	0.987
The work is unchanged	51 (54.3)	34 (54.0)	17 (54.8)	
The work has improved	27 (28.7)	18 (28.6)	9 (29.0)	
Are you feeling irritable?				
≤2	62 (66.0)	40 (63.5)	22 (71.0)	0.499
>2	32 (34.0)	23 (36.5)	9 (29.0)	
Mean (SD)	2.0 (1.2)	2.1 (1.1)	1.9 (1.3)	0.662
Do you feel lonely?				
≤2	74 (78.7)	51 (81.0)	23 (74.2)	0.452
>2	20 (21.3)	12 (19.0)	8 (25.8)	
Mean (SD)	1.7 (1.1)	1.3 (1.0)	1.8 (1.2)	0.278
Are you having problems falling asleep?				
≤2	55 (58.5)	35 (55.6)	20 (64.5)	0.407
>2	39 (41.5)	28 (44.4)	11 (35.5)	
Mean (SD)	2.3 (1.2)	2.3 (1.2)	2.1 (1.2)	0.477
Do you wake up in the night and have difficulties in getting back to sleep?				
≤2	44 (46.8)	27 (42.9)	17 (54.8)	0.274
>2	50 (53.2)	36 (57.1)	14 (45.2)	
Mean (SD)	2.5 (1.3)	2.6 (1.3)	2.4 (1.4)	0.488
Do you have nightmares?				
Yes	10 (10.6)	10 (15.9)	0 (0.0)	**0.028**
Are you afraid of being infected?				
≤2	31 (33.0)	18 (28.6)	13 (41.9)	0.195
>2	63 (67.0)	45 (71.4)	18 (58.1)	
Mean (SD)	3.1 (1.4)	3.3 (1.4)	2.7 (1.3)	**0.042**
Do you want to go out and go back to previous life?				
≤2	15 (16.0)	11 (17.5)	4 (12.9)	0.766
>2	79 (84.0)	52 (82.5)	27 (87.1)	
Mean (SD)	3.8 (1.3)	3.7 (1.3)	4.1 (1.2)	0.140
Do you think the pandemic has affected your life?				
No	14 (14.9)	10 (15.9)	4 (12.9)	0.769
Yes	80 (85.1)	53 (84.1)	27 (87.1)	

*p* values < 0.05 were considered statistically significant and reported in bold characters; ^1^ Percentages calculated on the number of participants with children (*n* = 69); ^2^ Percentages calculated on the number of participants with children <18 years (*n* = 20); ^3^ Percentages calculated on the number of participants with children ≥18 years (*n* = 49).

**Table 3:** Generalized linear model for perception in work changes, irritability and loneliness in the study population (N = 94) of home office workers during the COVID-19 pandemic.

Independent Variables	Work Changes		Irritability		Loneliness	
	B	P	B	P	B	P
Age (<40 years)	−1.75	0.057	0.55	0.518	−0.87	0.398
Gender (F)	−0.62	0.217	0.83	0.109	−0.85	0.128
Marital status (married/cohabitant)	−0.99	0.067	−0.43	0.427	−1.10	**0.040**
Education (graduated)	1.97	**0.002**	−0.86	0.138	0.44	0.501
Parenthood (yes)	45.63	0.999	24.77	0.999	2.11	1.000
Having children ≥18 years (no)	0.28	0.671	−0.68	0.329	−0.92	0.251
Children’s age affecting remote working (yes)	0.79	0.293	−1.71	**0.013**	−0.25	0.772
Physical diseases (no)	0.97	0.109	−0.74	0.188	−0.47	0.486
Medications (no)	−0.43	0.654	0.43	0.607	−0.30	0.735
SARS-CoV-2 infection risk exposure (no)	0.14	0.832	−1.67	**0.012**	−0.65	0.362
Contacts with SARS-CoV-2 infected subject (no)	0.76	0.436	0.68	0.468	−0.44	0.689
Satisfied with work performance (yes)	0.60	0.592	−3.03	**0.003**	−2.37	**0.029**
Time connected to the internet (more than before)	−1.44	**0.009**	1.31	**0.020**	0.55	0.345
Satisfied with the online services (yes)	0.06	0.964	0.23	0.892	20.34	0.999
Feeling tired after a video call (no)	0.74	0.119	−0.07	0.877	−0.50	0.304

*p* values < 0.05 were considered statistically significant and reported in bold characters.

**Table 4:** Generalized linear model for problems in falling asleep and nocturnal awakenings with difficulties to return to sleep; binary logistic regression for nightmare in the study population (N = 94) of home office workers during the COVID-19 pandemic.

Independent Variables	Falling Asleep Problems		Nocturnal Awakenings		Nightmares	
	B	P	B	P	OR	P
Age (<40 years)	0.76	0.321	−0.08	0.919	0.01	0.999
Gender (F)	0.83	0.078	0.69	0.149	1.01	0.999
Marital status (married/cohabitant)	0.05	0.923	−0.40	0.427	1.26	0.732
Education (graduated)	−0.87	0.139	−0.64	0.248	0.83	0.790
Parenthood (yes)	1.08	0.602	0.79	0.710	1.21	0.797
Having children ≥18 years (no)	−0.37	0.572	−0.10	0.877	0.98	0.980
Children’s age affecting remote working (yes)	0.31	0.637	−0.22	0.730	1.44	0.746
Physical diseases (no)	0.69	0.266	0.24	0.667	4.50	**0.030**
Medications (no)	−1.39	0.122	−0.57	0.503	1.22	0.859
SARS-CoV-2 infection risk exposure (no)	0.01	0.985	−0.76	0.217	1.66	0.553
Contacts with SARS-CoV-2 infected subject (no)	−1.08	0.213	−1.25	0.159	2.22	0.496
Satisfied with work performance (yes)	0.64	0.549	−1.63	0.086	2.00	0.999
Time connected to the internet (more than before)	1.82	**0.001**	2.04	**<0.001**	1.25	0.788
Satisfied with the online services (yes)	22.49	0.999	22.18	0.999	2.12	0.999
Feeling tired after a video call (no)	−0.73	0.099	−0.35	0.423	0.77	0.721

*p* values < 0.05 were considered statistically significant and reported in bold characters.

**Table 5:** Generalized linear model for fear of infection and willingness to go out and return to previous life; binary logistic regression for the perception that the pandemic has affected everyday life in the study population (N = 94) of home office workers during the COVID-19 pandemic.

Independent Variables	Fear of Infection		Willingness to Go Out and Return to Previous Life		Pandemic Influence on Everyday Life	
	B	P	B	P	OR	P
Age (<40 years)	0.27	0.716	−0.66	0.398	2.52	0.392
Gender (F)	0.69	0.126	−0.46	0.353	0.79	0.704
Marital status (married/cohabitant)	−0.78	0.115	−0.57	0.280	0.97	0.959
Education (graduated)	−0.01	0.989	−0.66	0.235	0.67	0.523
Parenthood (yes)	−1.21	0.598	−0.43	0.837	0.89	0.856
Having children ≥18 years (no)	0.70	0.256	0.36	0.555	0.57	0.502
Children’s age affecting remote working (yes)	0.39	0.511	−0.47	0.452	3.52	0.999
Physical diseases (no)	−0.77	0.159	0.98	0.077	0.64	0.516
Medications (no)	−0.25	0.793	−1.21	0.174	0.80	0.843
SARS-CoV-2 infection risk exposure (no)	−0.04	0.942	1.06	0.075	1.05	0.957
Contacts with SARS-CoV-2 infected subject (no)	0.65	0.427	0.85	0.287	0.01	0.999
Satisfied with work performance (yes)	−0.69	0.555	0.21	0.844	3.97	0.999
Time connected to the internet (more than before)	1.04	**0.039**	−0.35	0.519	0.23	**0.016**
Satisfied with the online services (yes)	−1.65	0.223	0.54	0.391	0.01	0.999
Feeling tired after a video call (no)	0.13	0.761	0.14	0.754	0.46	0.254

*p* values < 0.05 were considered statistically significant and reported in bold characters.

**Table 6:** Correlation matrix between considered outcomes regarding work performance, mood and sleep alteration in the study population (*N* = 94) of home office workers during the COVID-19 pandemic.

		1	2	3	4	5	6	7	8	9
1	Work changes	−	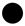	/	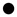	/	/	/	/	/
2	Irritability	−0.29	−	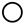	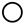	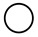	/	/	/	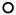
3	Loneliness	−0.13	0.29	−	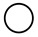	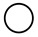	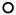	/	/	/
4	Falling asleep problems	−0.27	0.33	0.43	−	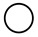	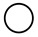	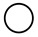	/	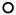
5	Nocturnal awakenings	−0.20	0.37	0.39	0.82	−	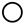	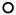	/	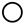
6	Nightmares	−0.06	0.09	0.24	0.36	0.35	−	/	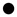	/
7	Fear of infection	−0.04	0.14	0.04	0.36	0.23	0.16	−	/	/
8	Willingness to go out and return to previous life	−0.10	0.17	−0.01	0.07	0.05	−0.15	0.03	−	/
9	Pandemic influence on everyday life	−0.11	0.24	0.13	0.24	0.27	0.05	0.17	−0.06	−

Black color indicates a negative correlation; white color indicates positive correlation. 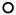: *p*-value < 0.05, 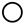: *p*-value < 0.01, 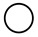: *p*-value < 0.001.

## Data Availability

The datasets generated and analyzed during the current study are available from the corresponding author on reasonable request.
